# TiO_2_ nanoparticles induce omphalocele in chicken embryo by disrupting Wnt signaling pathway

**DOI:** 10.1038/s41598-018-23215-7

**Published:** 2018-03-19

**Authors:** Shweta Patel, Sarmita Jana, Rajlakshmi Chetty, Sonal Thakore, Man Singh, Ranjitsinh Devkar

**Affiliations:** 1 Department of Zoology, Faculty of Science, The M.S. University of Baroda, Vadodara, India; 2 Department of Chemistry, Faculty of Science, The M.S. University of Baroda, Vadodara, India; 3 School of Chemical sciences, Central University of Gujarat, Gandhinagar, India

## Abstract

Titanium dioxide nanoparticles (TiO_2_ NPs) are among abundantly used metal oxide NPs but their interactions with biomolecules and subsequent embryonic toxicity in higher vertebrates is not extensively reported. Physicochemical interactions of TiO_2_ NPs with egg albumen reveals that lower doses of TiO_2_ NPs (10 and 25 µg/ml) accounted for higher friccohesity and activation energy but an increment in molecular radii was recorded at higher doses (50 and 100 µg/ml). FTIR analysis revealed conformational changes in secondary structure of egg albumen as a result of electrostratic interactions between egg albumen and TiO_2_ NPs. The morphometric data of chicken embryo recorded a reduction at all the doses of TiO_2_ NPs, but toxicity and developmental deformity (omphalocele and flexed limbs) were recorded at lower doses only. Inductively coupled plasma optical emission spectrometry (ICP-OES) confirmed presence of Ti in chicken embryos. mRNA levels of genes involved in canonical and non-canonical Wnt signaling were lowered following TiO_2_ NPs treatment resulting in free radical mediated disruption of lateral plate mesoderm and somite myogenesis. Conformational changes in egg albumen and subsequent developmental deformity in chicken embryo following TiO_2_ NPs treatment warrants detailed studies of NP toxicity at lower doses prior to their biomedical applications.

## Introduction

Nanotechnology is a rapidly expanding field, with a wide range of applications in communications, robotics, medicine, clothes, sporting goods, etc^1,2^. According to a recent survey, the number of nanotechnology-based consumer products available in the world market is more than 1800^3^. The increased use of nanomaterials is also under scrutiny due to their adverse effects on the environment, physiology and overall survival of organisms. Titanium dioxide nanoparticles (TiO_2_ NPs) are the most abundantly used nano metal oxides with their documented industrial uses in pigments and additives for paints, paper, ceramics, plastics, foods, and other products. The estimated worldwide production of TiO_2_ NPs is 10000 tons/year for 2011–2014 and 2.5 million metric tons/year by 2025^4^. Therefore, risk assessment studies have predicted that TiO_2_ NPs will be the most prevalent nanomaterials in environment^5^.

Cytotoxic potential of TiO_2_ NPs is well documented in a variety of cell lines. Oxidative DNA damage and apoptosis in HepG2 cells and in human epidermal cells^6^, apoptosis and/or necrosis in human astrocytoma (astrocytes-like) U87 cells^7^ and mitochondrial dysfunction in BRL 3A cells^8^ are some of the recent reports on cytotoxicity of TiO_2_ NPs. Toxicity of TiO_2_ NPs based on difference in their size has been documented in nematodes^9^ and earthworm^10^. The ability of TiO_2_ NPs to produce reactive oxygen species and surface charge are the reasons accredited for their toxicity^6,11^. Several engineered nanometals including TiO_2_ NPs have been known to persist in the food chain and move across trophic levels resulting in various forms of toxic manifestations^11^. Hence, their effect on reproductive performance and embryonic development cannot be ignored. Accelerated hatching of larvae and deformed embryos in zebrafish^12^ and histopathological changes in juvenile carp^13^ are few evidences on TiO_2_ NPs induced toxicity on embryonic and post-hatch development. Hatching inhibition and malformation of embryos of Abalone have been reported following TiO_2_ NPs exposure^14^. Also, prenatal exposure of TiO_2_ NPs in female rats impacts genes controlling brain development in offspring^15^ providing compelling evidences on systemic and developmental toxicity.

Chicken embryo is a sensitive and popularly used model for assessing developmental toxicity and teratogeny of various nanoparticles. Hence, chicken embryo was chosen as an experimental model in our study to assess the impact of TiO_2_ NPs on embryonic development. Other studies had reported developmental toxicity of graphite^16^, copper^17^, carbon^18^, platinum^19^, pristine graphene^20^ and silver^21^ nanoparticles on chicken embryo, but their *in ovo* physicochemical interactions with biomolecules such as egg albumen have not been taken into account. In the present study, we assess the interaction of TiO_2_ NPs with egg albumen and its subsequent impact on chicken embryonic development.

## Results

In DLS analysis, TiO_2_ NPs presented a single distribution with peak centered at 88.6 nm. The plot showed that the nanoparticles have a narrow size distribution with an average diameter of about 88.6 nm (Supplementary Figure S1).

### Physicochemical analysis

**Figure 1:**
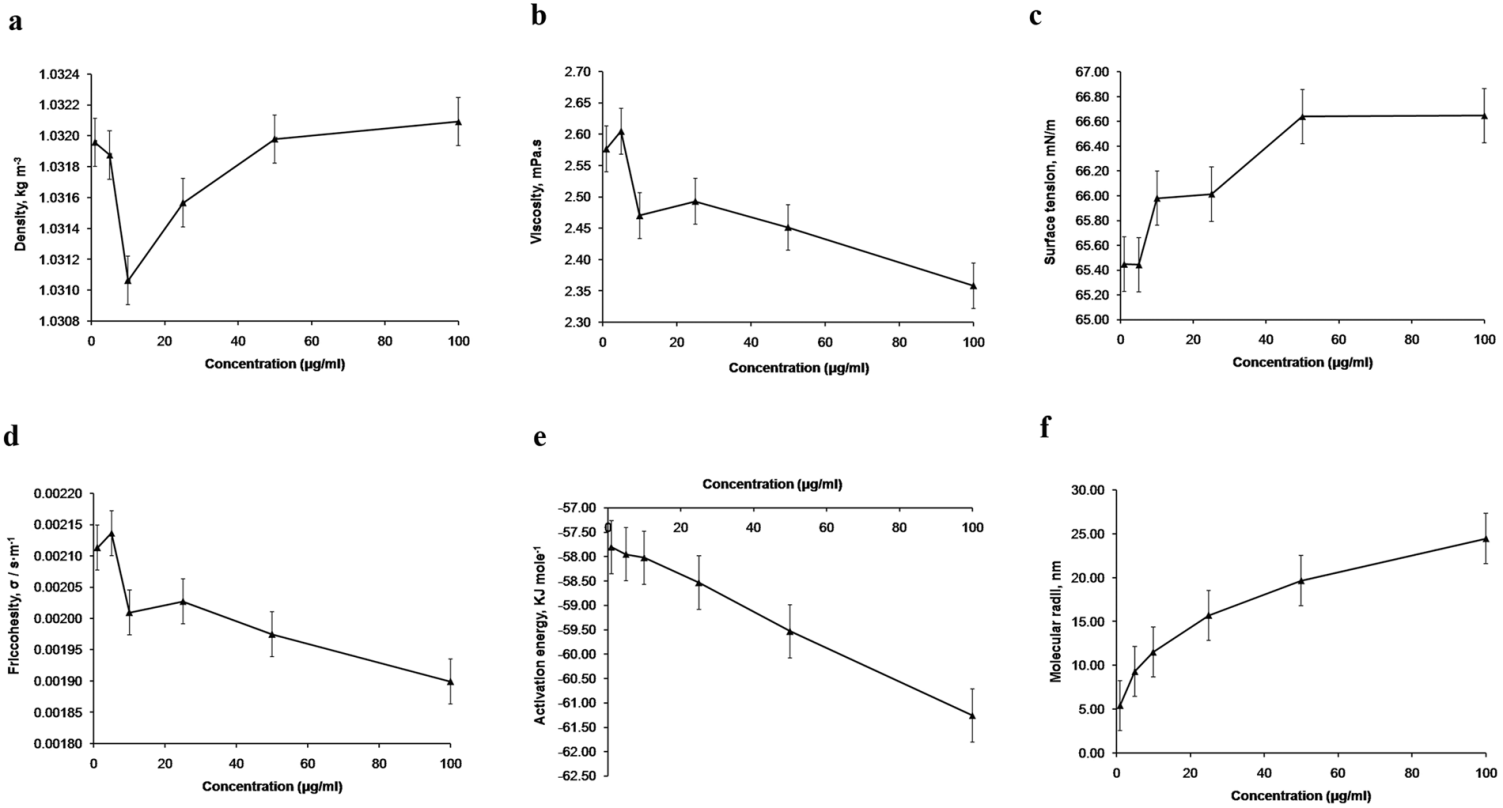
Physicochemical analysis of titanium dioxide nanoparticles and their interaction with egg albumen. Density, Viscosity, Surface tension, Friccohesity, Activation energy and Molecular radii of TiO_2_ NPs in albumen (**a**–**f**) respectively.

Results shown herein are quantification of interaction of peptide bonds with TiO_2_ NPs and alteration in the Lennard Jone potential that varies spontaneity and strength of interactive force. There was a decrement in density (1.031064 kg.m^−3^) of TiO_2_ NPs + albumen at 10 µg/ml, whereas, 25, 50 and 100 µg/ml recorded steadily ascending values (1.031567, 1.031979 and 1.032092 kg.m^−3^ respectively) (Fig. 1a and Supplementary Table S2). Lower concentrations of TiO_2_ NPs (1 and 5 µg/ml) recorded higher viscosity indices (2.57 and 2.60 mPa.s). The viscosity indices of 10, 25 and 50 µg/ml doses were comparable to each other (2.47, 2.49 and 2.45 mPa.s) but, 100 µg/ml dose accounted for a decline in viscosity (2.35 mPa.s) (Fig. 1b and Supplementary Table S2). Indices of surface tension showed an increase at 10 µg/ml concentration of TiO_2_ NPs (65.98 mN.m^−1^) as compared to 1 and 5 µg/ml concentrations (65.45 and 65.44 mN.m^−1^ respectively). However, 25, 50 and 100 µg/ml doses recorded a steady increment in surface tension (66.01, 66.64 and 66.65 mN.m^−1^) (Fig. 1c and Supplementary Table S2). Friccohesity indices showed a decline at 10 µg/ml TiO_2_ NPs (0.002009 s.m^−1^) as compared to 1 and 5 µg/ml concentrations (0.002113 and 0.002136 s.m^−1^ respectively). A steady decline in friccohesity (0.002027, 0.001974 and 0.001899 s.m^−1^ at 25, 50 and 100 µg/ml doses respectively) was also observed in this study (Fig. 1d and Supplementary Table S2). A dose dependent decline in activation energy was recorded from 1–100 µg/ml doses with −57.81 KJ.mole^−1^ as the highest value and −61.26 KJ.mole^−1^ as the lowest value respectively (Fig. 1e and Supplementary Table S2). An increase in molecular radii (5–11.53 nm) was observed at 1–10 µg/ml TiO_2_ NPs. Further, a steady increase in molecular radii (15.69, 19.66 and 24.45 nm) was observed at 25, 50 and 100 µg/ml doses respectively (Fig. 1f and Supplementary Table S2).

### Spectroscopic characterization

**Figure 2:**
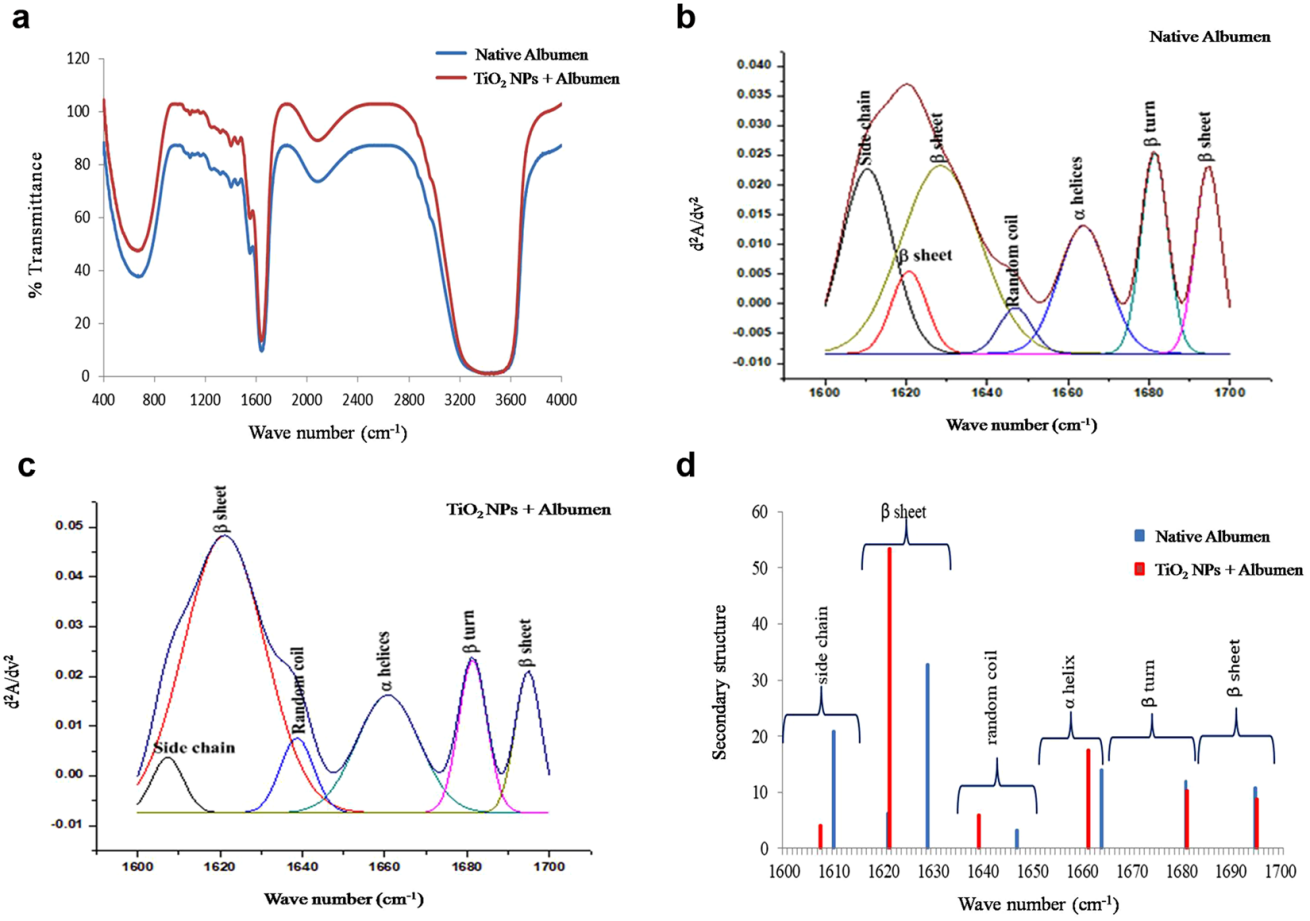
FTIR spectra of native egg albumen and TiO_2_ NPs + Albumen. (**a**) Entire spectral range from 400–4000 cm^−1^; (**b**–**d**) Gaussian curve fitting of secondary derivative of Native Albumen and TiO_2_ NPs + Albumen with Comparative Secondary structure elements from FTIR spectra.

Comparative FTIR spectra (400–4000 cm^−1^) of native albumen and TiO_2_ NPs + albumen depicting amide A (around 3400 cm^−1^), amide B (about 3090 cm^−1^), amide I and II (region between 1600–1700 cm^−1^) domains are shown in Fig. 2a. The broad peak at 3591 and 3434 cm^−1^ in the amide A region of native albumen corresponds to the H-O-H asymmetric and symmetric stretching respectively whereas TiO_2_ NPs + albumen recorded a shift in H-O-H stretching peak to 3478 cm^−1^. Also, a shift in peak from 2071 cm^−1^ (in albumen) to 2083 cm^−1^ (TiO_2_ NPs + albumen) was recorded. The amide I and II secondary fingerprint regions (in albumen) recorded two peaks at 1651 cm^−1^ and 1642 cm^−1^ but in TiO_2_ NPs + albumen a peak was recorded at 1641 cm^−1^. A peak in the amide III region was recorded at 1243 cm^−1^ in albumen whereas, TiO_2_ NPs + albumen recorded a shift to peak 1551 cm^−1^. Also, peaks at 1457 and 1451 cm^−1^ in albumen and TiO_2_ NPs + albumen respectively are due to –CH_2_ scissoring vibration whereas, a peak at 675 cm^−1^ in TiO_2_ NPs + albumen corresponds to Ti-O vibrational mode of TiO_2_ NPs. Results obtained in deconvoluted Gaussian fitted spectra (Fig. 2b,c) and integrated peak areas of secondary-derivative structure element (Fig. 2d and Supplementary Table S3) and in albumen and TiO_2_ NPs + albumen showed that albumen was mainly composed of side chain (1610 cm^−1^, 20.93%) inter or intramolecular β sheet (1621–1629 cm^−1^, 39.11%) closely followed by α helices (1664 cm^−1^, 13.94%) with minor proportions of β turns (1681 cm^−1^, 11.95%) and β sheet (1695 cm^−1^, 10.76%). After interaction with TiO_2_ NPs, a decrease in side chain (1607 cm^−1^, 4.11%), β turns (1681 cm^−1^, 10.31%) and β sheet (1695 cm^−1^, 8.81%) and an increase in inter or intramolecular β sheet (1621cm^−1^, 53.39%) and α helices (1661 cm^−1^, 17.46%) was recorded.

### Natality and Morphometry of Chick embryos

**Figure 3:**
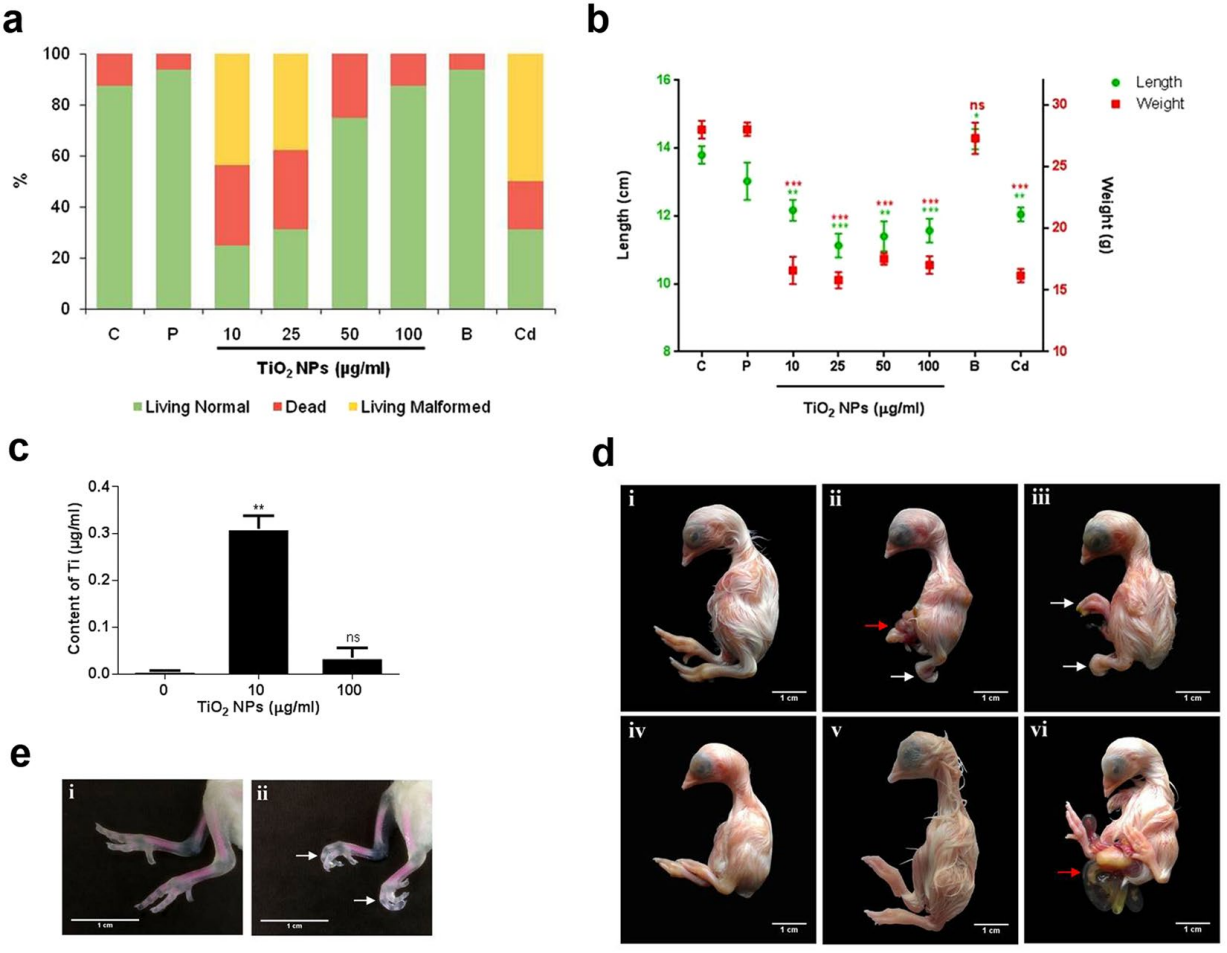
TiO_2_ NPs induces developmental deformities in chicken embryo. (**a**) Percentages of living normal, dead and malformed embryos in 19-day-old chick embryos. (**b**) Mean body weight (g) and lengths (cm) of living 19-day-old chick embryos in control and treated groups. (**c**) Concentrations of Ti in embryos of control and TiO_2_ NPs-treated group (4 day old) by ICP-OES. (**d**) Chicken-embryo development after 19 days of incubation, control (i), 10–100 µg/ml TiO_2_ NPs-treated groups (ii,iii,iv), 10 µg/ml TiO_2_ Bulk (v) and 10 µg/ml cadmium chloride (vi), (Scale bar: 1 cm). Embryos treated with 10 µg/ml TiO_2_ NPs (ii) exhibits omphalocele (red arrow) & flexed limbs (white arrow). (**e**) Photographs showing endoskeleton (bone and cartilage) of 19-day-old chick embryos, control (i) and TiO_2_ NPs–treated (ii) stained with Alizarin red S and alcian blue staining. TiO_2_ NPs treated (10 µg/ml) embryos exhibits flexed digits (arrow). The data are expressed as Mean ± SD. Statistical analysis was done by one way ANOVA, *p ≤ 0.05, **p < 0.01, ***p < 0.001, ns- not significant, C- control, P-placebo, B- TiO_2_ Bulk (10 µg/ml), Cd- cadmium chloride (10 µg/ml).

Lower doses (10 and 25 µg/ml) of TiO_2_ NPs treatment accounted for 12.5% and 25% viable embryos respectively on 19^th^ day of incubation. Also, 56.25% and 43.75% embryos were found to be malformed at 10 and 25 µg/ml doses. However, higher doses (50 and 100 µg/ml) recorded viable embryos ranging between 75–87.5% (Fig. 3a). Morphometry of the embryos (whole weight and length) recorded significant decrement at all the doses (10–100 µg/ml) (Fig. 3b). Whole weights of liver, brain and heart showed non-significant decrement at all the said doses (Supplementary Table S4).

### ICP-OES analysis of embryos

After 4-days of TiO_2_ NPs treatment, the contents of Ti in the chick embryo were measured by ICP-OES. Significantly high levels of Ti was detected (3 times increase) in embryos of eggs treated with 10 µg/ml TiO_2_. But, higher dose (100 µg/ml) accounted for a moderate non-significant content of Ti in embryos (Fig. 3c).

### Deformity

Control and TiO_2_ NPs treated chick embryos were examined as per Hamburger-Hamilton standard that revealed presence of flexed limbs at 10 and 25 µg/ml doses of TiO_2_ NPs (Fig. 3d). Also, omphalocele (ventral body wall defect) was observed at 10 µg/ml dose. These deformities were not seen at any of the higher doses (50 and 100 µg/ml). Further confirmation of flexed limbs of 10 and 25 µg/ml TiO_2_ NPs treated embryos was obtained by alcian blue- alizarine red staining (Fig. 3e).

### Expression of Wnt signaling genes

**Figure 4:**
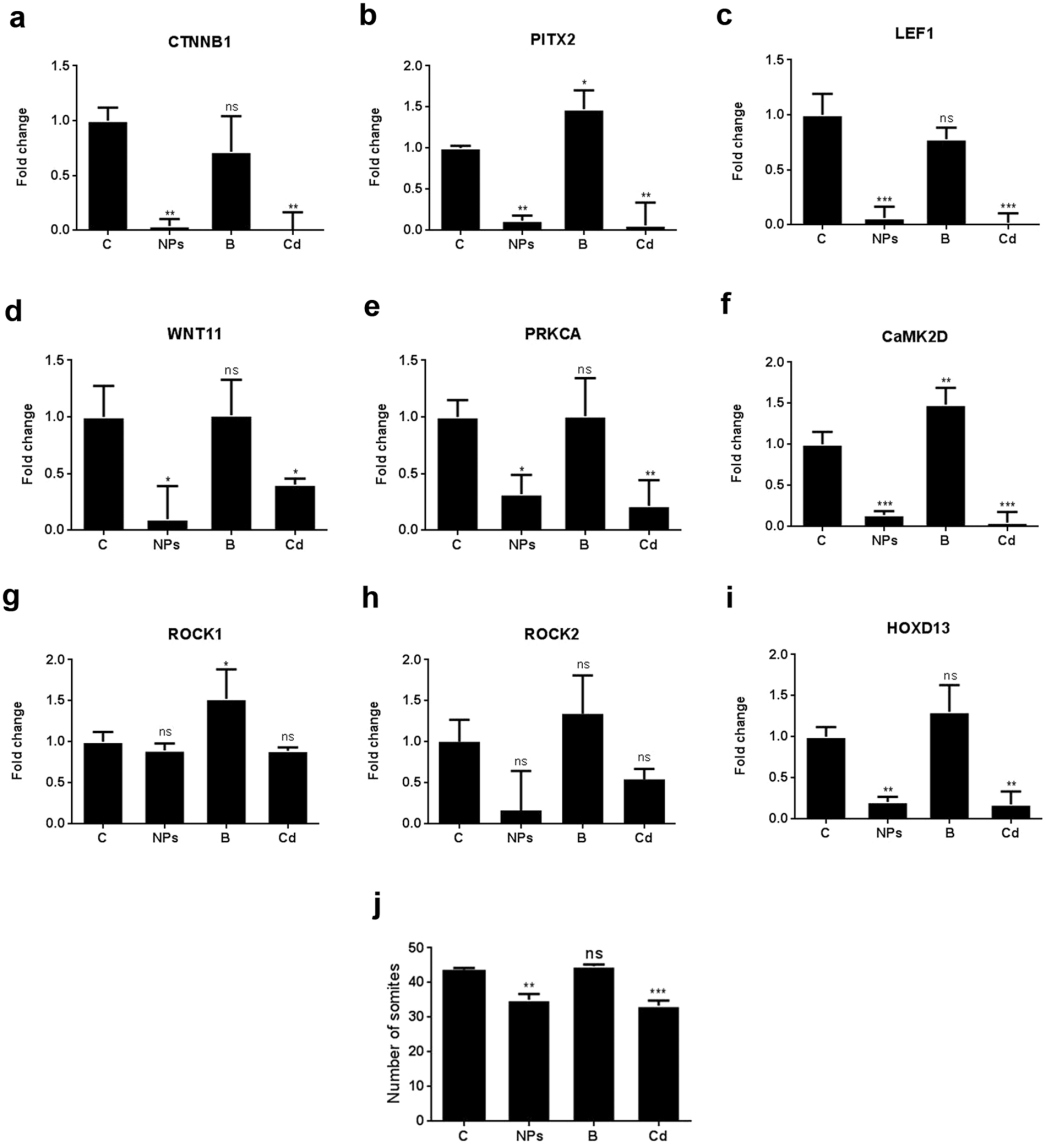
Expression of Wnt signaling pathway-related genes in TiO_2_ NPs-treated chicken embryos. The expression of Wnt signaling pathway-related genes (**a**–**h**) including CTNNB1, PITX2, LEF1, WNT11, PRKCA, CAMK2D, ROCK1 and ROCK2 and (**i**) limb development gene HOXD13 was analyzed using reverse transcription polymerase chain reaction (RT-PCR) in control and TiO_2_ NPs-treated embryos (n = 3), 4 h after treatment in shell-less culture at 60 h. All the Wnt signaling pathway related genes and limb development gene were downregulated in TiO_2_ NPs-treated embryos compared to those of control embryos. *p < 0.05, **p < 0.01, ***p < 0.001, ns = not significant. (**j**) Somite numbers in control and TiO_2_ NPs-treated embryos 24 h after treatment in shell-less culture (HH-23). There is a reduction in the number of somites in TiO_2_ NPs-treated embryos as compared to the control embryos. The data are expressed as Mean ± SD. Statistical analysis was done by one way ANOVA followed by Dunnett’s test. *p < 0.05, **p < 0.01, ***p < 0.001, ns = not significant.

RT-PCR analysis was performed to assess the effect of TiO_2_ NPs on expression of key genes of canonical (CTNNB1, PITX2 and LEF1), non-canonical Wnt/Ca^2+^ (WNT11, PRKCA and CAMK2D) and Planar Cell Polarity (ROCK1 and ROCK2) pathways associated with Wnt signaling. Expression levels of genes of canonical pathway (CTNNB1, PITX2 and LEF1) were downregulated significantly in embryos treated with TiO_2_ NPs (10 µg/ml). A Similar trend of significant decrement was also observed in cadmium treated embryos, whereas, TiO_2_ bulk treatment could not manifest any significant change (Fig. 4a–c). Expression levels of key genes of non-canonical Wnt/Ca^2+^ Wnt signaling (WNT11, PRKCA and CAMK2D) showed significantly lowered expression levels following TiO_2_ NPs or cadmium treatment. However, the TiO_2_ bulk treatment showed non-significant changes in the expression levels of the said genes (Fig. 4d–f). mRNA expression of key genes of Planar Cell Polarity pathway (ROCK1 and ROCK2) accounted for non-significant decrement following TiO_2_ NPs or cadmium treatment. TiO_2_ bulk treatment accounted for moderately significant increment in ROCK1 expression and non-significant increment in expression of ROCK2 (Fig. 4g and h). Expression levels of HOXD13 showed significantly lowered expression levels following TiO_2_ NPs or cadmium treatment, whereas, the TiO_2_ bulk treatment showed non-significant changes (Fig. 4i).

### Somite development

It was observed that 10 µg/ml dose of TiO_2_ NPs accounted for 20% decrement in the number of somites after 24 h which was comparable to that of the cadmium treated group, whereas, TiO_2_ bulk treatment could not manifest any significant change (Fig. 4j).

## Discussion

Nanomaterials have been reported to interact with protein molecules in unique ways and form a ‘protein corona’ that alters its physicochemical identity and affect its bio-distribution, kinetics and subsequent toxicity^22^. A previous study in our lab had shown that TiO_2_ NPs interact with protein components of RPMI-1640 and result in higher indices of intermolecular interaction^23^. Egg albumen is reservoir of protein in an avian egg that meets the nutritional requirements of an embryo. Besides egg shell, shell membrane and chorio-allantoic membrane; egg albumen also regulates the trafficking of exogenous elements by acting as a natural biological barrier^24^. In the present study, a dose dependent increase in density of albumen was observed following addition of TiO_2_ NPs. But, relatively lowest density observed at 10 µg/ml hints at effective dispersion of TiO_2_ NPs in egg albumen. Higher intermolecular forces and cohesion are the key factors that determine viscosity and surface tension of liquids. In our study, a dose dependent decrement in viscosity and a reciprocal increment in surface tension are in support of our claim that higher intermolecular forces are as a result of higher concentration of TiO_2_ NPs. Friccohesity is a product of frictional and cohesive forces within similar (protein-protein) and dissimilar (protein-nanoparticle) molecules^25^. A dose dependent decrement in friccohesity suggests weaker inter conversion between cohesive and frictional forces implying towards a stronger nanoparticles-egg albumen interaction. Also, a decrement in activation energy is an indicator of increased proportion of collision/chemical reaction between the test compounds^23^. Relatively higher indices of activation energy recorded in TiO_2_ NPs + albumen (10 µg/ml) is suggestive of more quantum of interaction between TiO_2_ NPs and egg albumen. Molecular radii play an important role in dispersion of nanometals and its impact on biological systems. A dose dependent increment in molecular radii resulting due to TiO_2_ NPs-egg albumen interactions implies towards formation of nanoparticle agglomerates at higher doses.

TiO_2_ NPs + albumen recorded a shift in H-O-H stretching peak to 3478 cm^−1^ confirming interaction between TiO_2_ NPs and albumen. Further a shift in peak from 2071 cm^−1^ (in albumen) to 2083 cm^−1^ (TiO_2_ NPs + albumen) is attributable to interaction between C-O and amide groups of amino acids present in albumen. Peaks observed in the amide I and II secondary fingerprint region (in albumen) at 1651 cm^−1^ and 1642 cm^−1^ are attributable to C=O stretching and H-O-H bending respectively. However, a minor shift in C=O stretching and depletion of H-O-H bending (at 1641 cm^−1^) was possibly on account of electrostatic interaction due to Vander Waal forces taking place between albumen and TiO_2_ NPs. Peak in amide III region (at 1243 cm^−1^) in albumen occurs due to N-H bending and C-N stretching of amino groups but, TiO_2_ NPs + albumen recorded a shift (at 1551 cm^−1^) from amide III to amide II region. This shift also portrays major conformational changes in secondary components (α helices and β sheet) of proteins possibly due to their interaction with TiO_2_ NPs. Fourier-self deconvolution approach was employed to assess secondary conformational changes in amide I and II region. Venyaminov and Kalnin^26^ had reported that amide peak at 1610 ± 4 corresponds to NH bending of CO-NH_2_ bond in glutamine. In our study, amide peak at 1610 cm^−1^ in TiO_2_ NPs + albumen indicates possible deformity of glutamate in egg albumen. Role of glutamate in nutrition and metabolism is well reported^27^ and hence impact of structurally altered glutamate on developing chicken embryo is postulated herein. Further, an increase in β sheet and α helices in TiO_2_ NPs + albumen are possibly due to TiO_2_ NPs mediated conformational changes, formation of aggregates or amyloids with protein moieties in egg albumen. These findings are the first to showcase interaction of TiO_2_ NPs with egg albumen and the said physicochemical alterations.

Interaction of TiO_2_ NPs with egg albumen prompted us to assess its impact on embryonic development using chicken egg as a model. Significant reduction in morphometric indices (body weight and length) and higher percentage mortality was recorded in developing chicken embryos at lower doses of TiO_2_ NPs (10 µg/ml). But, the higher doses of TiO_2_ NPs (50 or 100 µg/ml) failed to elicit a dose-dependent toxicological response possibly because TiO_2_ NPs underwent physicochemical alterations as evidenced by relatively higher indices of density, viscosity and friccohesity coupled with lower activation energy hinting at formation of NP agglomerates. Whereas, lower extent of NP-egg albumen interactions observed at 10 µg/ml of TiO_2_ NPs was instrumental in its effective bio-distribution and manifested said toxicity. Percentage mortality of bulk TiO_2_ treated chicken embryos was comparable to that of higher doses (50 or 100 µg/ml) of TiO_2_ NPs thus providing conclusive evidence that an altered physicochemical identity of NPs failed to induce a dose dependent toxicity in chicken embryos. Nanoparticles are known to cross biological barriers like the blood brain barrier and blood placenta barrier^28^. The results obtained herein indicate that the TiO_2_ NPs could cross biological barriers within an avian egg and reach the embryo. The same was confirmed by ICP-OES studies that revealed presence of higher levels of TiO_2_ NPs in the embryonic tissue at the lower dose (10 µg/ml).

Omphalocele is a ventral body wall defect and is accompanied by herniation of midgut into the abdominal cavity, failure in fusion of the anterior abdominal wall with 1/3000 frequency of occurrence in human population^29^. Teratogenic agents such as cadmium^30^, specific radiations^31^, fungal toxins^32^, etc. are known to induce omphalocele in various animal models. However, no known nanomaterials have been reported to induce omphalocele. Wnt signaling pathway has been implicated in various events of embryonic development such as cell differentiation, survival, migration, proliferation, adhesion and somite formation^33^. Canonical Wnts relay their signal via ß-Catenin pathway that control cell fate determination^33^. Whereas, the non-canonical Wnt signaling either through Wnt/Ca^2+^ pathway or planar cell polarity pathway that controls cell adhesion and movement^33^. Results obtained herein were compared with cadmium induced omphalocele chicken embryo model^30^. PITX2, a bicoid-type homeodomain transcription factor, has known to be regulated by ß-Catenin dependent Wnt pathway^34^. In the Wnt/ß-Catenin pathway, the accumulation of ß-Catenin in the nucleus converts DNA-binding factor, lymphoid enhancing factor-1 (LEF1), to a transcriptional activator and is regulated through direct physical interaction with PITX2 and ß-Catenin^35^. In this study, downregulation in expression levels of CTNNB1, PITX2 and LEF1 following TiO_2_ NPs treatment (10 µg/ml) could be a key factor in the disruption of somite myogenesis by inhibiting Wnt/ß-Catenin pathway. It has been postulated that cells from somites migrate into the parietal layer of lateral plate mesoderm (LPM) to assist in forming the lateral body folds^36^. PITX2 is known to regulate cell survival^37^ and its downregulation may induce abnormal apoptosis in the somite and LPM that could further interfere with the movement of the lateral body wall folds^29^. These results justify the decrement in somite count obtained in our study following TiO_2_ NPs treatment (10 µg/ml). WNT11, a member of the noncanonical Wnts, is an important epithelialization factor acting on the dermomyotome whereas, PRKCA and CaMK2D control actin-cytoskeleton organization and cell contractility^33,38^. Previous studies had implicated PRKCA and CaMK2D (activated by WNT11) in the regulation of cell-cell adhesion molecules (CAMs) such as cadherins. The resultant linkages between E-cadherin and actin filaments reinforce the cell-cell junctional connection^39^. In our study, downregulation of WNT11, PRKCA and CaMK2D genes after TiO_2_ NPs treatment (10 µg/ml) possibly interfered with actin-cytoskeleton organization, cell movement and cell adhesion, thus disrupting noncanonical Wnt/Ca^2+^ signaling that resulted in omphalocele. Rho kinases (ROCK) are involved in the regulation of various cellular functions (contraction, adhesion, migration, proliferation and apoptosis) including tissue closure during embryonic development. ROCK1 and ROCK2 mediate signaling from Rho to the actin cytoskeleton in the Wnt non-canonical pathway^40^. ROCK1 knockout (KO), ROCK2 KO, and ROCK1/2 double heterozygous mice has been reported to exhibit omphalocele phenotype due to disorganization of actin filament in the epithelial cells of umbilical ring^41^. Downregulation of ROCK genes following TiO_2_ NPs treatment possibly disrupted actomyosin assembly, resulting in the failure of ventral body wall closure resulting in omphalocele. Defects in ventral body wall closure and omphalocele has also been reported with accompanying limb deformities in genetically modified experimental models^42^. *Hox* genes are important regulators of limb pattern in vertebrate development, HOXD13 misexpression in the hindlimb results in shortening of the long bones, including the femur, the tibia, the fibula and the tarsometatarsals^43^. In our study, significantly lowered expression of HOXD13 in TiO_2_ NPs treated embryos corroborate with the observed omphalocele. Cadmium is known to use Ca^2+^ ion channels and membrane transporters to enter in to the cells of a developing embryo. Further, it disrupts lateral plate mesodermal cells and induces omphalocele^30^. Therefore, cadmium treated chicken embryos were used as a disease control in our study wherein; expression levels of key genes of the Wnt signaling pathways were comparable to TiO_2_ NP treated embryos. TiO_2_ NPs are also known to cause free radicals induced cellular damage^6^. Free radical induced disruption of lateral plate mesoderm and somite myogenesis culminating in omphalocele in TiO_2_ NPs treated chicken embryos is hypothesized in our study.

Besides their widespread industrial use, TiO_2_ NPs have gained prominence in biomedical applications due to their long term photostability, superior biocompatibility, catalytic efficiency and a strong oxidizing power^44,45^. Photodynamic therapy for cancer, cell imaging, genetic engineering, drug delivery and biosensors are some of the reported biomedical applications of TiO_2_ NPs^44,45^. Also, their use in diagnosis of cardiovascular diseases, diabetes mellitus, cancer and orthopaedic disorders underlines their prominence. But, omphalocele formation only at sub lethal (lower) concentrations reported herein raises concerns of toxicity benchmarks impacting foetal development. Hence, it raises an urge to study interactions of nanoparticles with biomolecules vis-à-vis particle size or surface modifications prior to their use in diagnostics or biomedical applications.

## Conclusion

Nanometal oxides witness a wide range of biomolecules in a physiological environment that can alter their behavior and responses. In the present study, TiO_2_ NPs were found to interact with egg albumen as evidenced by changes in their proteinic secondary structure. These interactions could possibly allow TiO_2_ NPs to traverse the biological barriers (shell membrane and CAM) within chicken egg and affect the growth and development of embryos and cause malformations like omphalocele and flexed limbs. Also, the observed mortality and significant decrement in morphometry (whole weight and length) are attributable to TiO_2_ NPs-albumen interactions. Omphalocele formation in TiO_2_ NPs treated groups is possibly due to the disruption of somite myogenesis as evidenced by alterations in expression of key genes of Wnt signaling pathway. Hence, use of TiO_2_ NPs in diagnostics and therapy warrants a detailed research in embryos by taking into account its particle size, surface modifications and interaction with biomolecules.

## Materials and Methods

### Availability of Data and Materials

The datasets supporting the conclusions of this article are included within the article.

### Nanoparticles

Titanium (IV) oxide nanopowder (TiO_2_ NPs, mixture of Anatase and rutile, Cat. no. 634662, particle size <100 nm, 99.5% purity) was procured from Aldrich (St. Louis, MO, USA). TiO_2_ NPs (1 mg/ml) were suspended in water and probe sonicated (LMUC-4, Labman scientific instruments Pvt. Ltd., Kolkata, India) for 30 min. After sonication, the particle size distribution was measured using a 90 plus DLS (Dynamic light scattering) unit from Brookhaven (Holtsville, USA).

### Physicochemical analysis

The physicochemical properties such as density, viscosity, surface tension, activation energy, friccohesity and molecular radii were assessed in absence or presence of TiO_2_ NPs in egg albumen (freshly collected). TiO_2_ NPs were suspended in egg albumen at 1, 5, 10, 25, 50 and 100 μg/ml concentrations. Density of TiO_2_ NPs in water and egg albumen were determined with Anton Paar Density and Sound velocity Meter (DSA 5000 M). Density was calculated using equation 1:

1 $${\rm{\rho }}={\rm{\rho }}^\circ +{{\rm{S}}}_{{\rm{\rho }}}{\rm{m}}+{{\rm{S}}^{\prime} }_{{\rm{\rho }}}{{\rm{m}}}^{2}$$

(ρ° at m → 0 is limiting density, Sρ is the 1^st^ slope)

Viscosity was measured as viscous flow times (VFT) using Borosil Mansingh Survismeter^23^ at physiological temperature of 37 °C (LAUDA ALPHA RA 8 thermostat) and calculated by equation 2:

2 $${\rm{\eta }}=(\frac{{\rm{t}}}{{{\rm{t}}}_{^\circ }})(\frac{{\rm{\rho }}}{{{\rm{\rho }}}_{^\circ }}){{\rm{\eta }}}_{^\circ }$$

(η_°_ is viscosity of water and t_°_, t are flow times of solvent and mixtures respectively)

The η data were regressed with following equation 3:

3 $${\rm{\eta }}={\rm{\eta }}^\circ +{{\rm{S}}}_{{\rm{\eta }}}{\rm{m}}$$

(η° atm → 0 is limiting viscosity; S_η_ is the 1^st^ degree slope).

Surface tension was measured by counting pendent drop numbers (PDN) using Borosil Mansingh Survismeter and calculated by equation 4:

4 $${\rm{\gamma }}=(\frac{{{\rm{\eta }}}_{^\circ }}{{\rm{\eta }}})(\frac{{{\rm{\rho }}}_{^\circ }}{{\rm{\rho }}}){{\rm{\gamma }}}_{^\circ }$$

(γ_°_ is surface tension of water, η_°_ and η are pendent drop numbers of medium and solutions respectively)

The γ data were regressed for limiting values γ° at m → 0 with following equation 5:

5 $${\rm{\gamma }}={\rm{\gamma }}^\circ +{{\rm{S}}}_{{\rm{\gamma }}}{\rm{m}}$$

(γ° is limiting surface tension, and S_γ_ is the 1^st^ degree slope)

Friccohesity was calculated using Mansingh equation 6^46^:

6 $$\sigma ={{\rm{\sigma }}}_{\circ }[(\frac{t}{{t}_{\circ }}\pm \frac{B}{t})(\frac{\eta }{{\eta }_{\circ }}\pm 0.0012(1-p))]$$

(σ is friccohesity, t and t_°_ are the sample and solution viscous flow times respectively, η_°_ and η are the pendant drop numbers of medium and solutions respectively)

Reference friccohesity was calculated by equation 7

7 $${{\rm{\sigma }}}_{\circ }={{\rm{\eta }}}_{\circ }{/{\rm{\gamma }}}_{\circ }$$

where, η_°_ and γ_°_ are the viscosity and surface tension of references respectively.

For activation energy, the partial molar volume V_2_ was calculated with following equation 8:

8 $${{\rm{V}}}_{2}=[\frac{1000({\rho }^{\circ }-\rho )}{m{\rho }^{\circ }\rho }]+\frac{M}{\rho }$$

(M is molar mass, ρ° is density of water and ρ is density of solution)

The V_1_ for water or albumen at 37 °C is calculated with equation 9:

9 $${{\rm{V}}}_{1}=\frac{M}{\rho }$$

V_1_ and V_2_ are used for calculating activation energy by using equation 10:

10 $${{{\rm{\Delta }}\mu }_{1}}^{\ast }={\rm{RT}}\,\mathrm{ln}(\frac{{\eta }_{\circ }{V}_{1}}{hN})$$

(Δµ_1_^*^ is activation energy of water or albumen, R is gas constant, h is Planck constant and N is Avogadro number (6.023 × 10^23^). Activation energy (Δμ_2_^*^J/mol) was calculated by using equation 11:

11 $${{{\rm{\Delta }}\mu }_{2}}^{\ast }\,=\,{\rm{\Delta }}{{\mu }_{1}}^{\ast }-[(\frac{RT}{{V}_{2}})((1000\eta )-({V}_{1}-{V}_{2}))]$$

Molecular radii r (nm) is calculated by using equation 12:

12 $${\rm{r}}=\sqrt[3]{\frac{3\varphi }{4\pi Nc}}$$

(ϕ is volume fraction of water or albumen entangled with NPs, N is Avogadro number, c is concentration and π is constant).

Each parameter was measured in triplicates.

### Spectroscopic characterization

Protein-nanoparticle interaction was identified by Fourier transform infrared (FTIR) spectra (PerkinElmer spectrum 65 series, PerkinElmer, Inc., MA, USA). Sample was prepared by making pellet of 1.5 to 2 mg of sample mixed with 200 mg KBr (AR, Sigma, USA) in the KBr press machine (model Mp-15) at 5 kg/cm^2^ pressure for 2 min. After taking background scan, samples were analyzed at 400–4000 cm^−1^. FTIR direct-transmittance spectroscopy (KBr) was used to indicate the degree to which oxygen groups were removed and the IR absorption of water from the air was mostly eliminated. Each measurement was repeated in triplicates to minimize the error.

### Chicken Embryo model and experimental groups

The experimental protocol (MSU-Z/IAEC/03-2017) was approved by the Institutional Animal Ethical Committee (IAEC) and the Committee for the Purpose of Control and Supervision of Experiments on Animals (827/GO/Re/S/04/CPCSEA). Fertilized eggs (55 ± 2.1 g) of White leghorn (*Gallus gallus domesticus*) were obtained from Shakti hatcheries, Sarsa, Gujarat, stored for 2 days at 12 °C and then incubated under standard conditions (37.5 °C, humidity 60%) for 48 hours and guidelines of Committee for the Purpose of Control and Supervision of Experiments on Animals (CPCSEA) were hereby followed for all the experiments conducted on the chicken embryo. The procedures for *in ovo* experimentation were as per the standard operating protocols of our laboratory. Candling was done to confirm the fertility of eggs and unfertile eggs were discarded. Eggs were randomly divided into six groups of 16 eggs/group viz. control (untreated), placebo (treated with PBS) and TiO_2_ treated (10, 25, 50 and 100 µg/ml) groups. TiO_2_ NPs powder was suspended in saline (1 mg/ml) and sonicated (LMUC-4, Labman scientific instruments Pvt. Ltd. Kolkata, India) for 30 min. Further, TiO_2_ NPs were diluted to 10, 25, 50 and 100 µg/ml doses. Test samples were injected *in ovo* (0.3 ml/egg) in airspace using sterile 1 ml tuberculin syringe and incubated for 18 days.

### Autopsy, morphometry and staining

On 19^th^ day, the experiment was terminated by opening the eggs and the viable embryos were weighed and decapitated. The morphology of the embryos was examined according to the Hamburger and Hamilton^47^ standards and ratios of live vs. dead/malformed were recorded. Deformities of the head, limbs, body and tail were observed under a dissecting microscope and photographed with a digital (Nikon coolpix p900) camera. Two embryos per group were processed for Alizarine-alcian blue staining^48^. Briefly, skin and viscera was removed and embryos were fixed in 96% ethanol for 3 days followed by acetone for 2 days. Later, embryos were then rinsed in ethanol for 1–2 h and stained with Alizarine-alcian blue stain (0.015% Alcian blue, 0.005% Alizarin red in 70% ethanol, 20% acetic acid and 10% dH_2_O) at 37 °C for 4 h. Embryos were rinsed in ethanol and running tap water for one hour each and muscles were cleared in an aqueous solution of 1% potassium hydroxide. De-staining of embryos was done in a graded series of glycerol/potassium hydroxide (20% glycerol/0.8% KOH, 50% glycerol/ 0.5% KOH and 80% glycerol/0.2% KOH respectively) and stored in 100% glycerol.

### Inductively coupled plasma optical emission spectroscopy (ICP-OES)

Four days old control and treated eggs (three per group) were opened and embryos were digested overnight at 40 °C in 6 ml of conc. nitric acid and 3 ml of hydrogen peroxide. Contents were heated in an oven (110 °C) for 2 h, cooled at room temperature and diluted with 11 ml of dH_2_O. The concentrations of nanosized particles in the embryo were quantified by ICP-OES^49^.

### Shell-less culture and dosing

In a separate set of experiment, procured eggs were incubated for 60 h in standard conditions and later were explanted into shell-less culture as per Dugan, *et al*.^50^. The embryos were divided into four groups of six eggs each viz control (50 µl PBS), positive control (50 µl of 50 µM CdCl_2_), TiO_2_ NPs treated (50 µl of 10 µg/ml) and bulk TiO_2_ (50 µl of 10 µg/ml). Dosing was done directly on blastodisc using a micropipette and embryos were incubated for 4 h or 24 h.

### Autopsy, RNA isolation and qPCR study

Developing embryonal discs (three per group, HH 17; whole embryo) were transferred in RNA later solution (Invitrogen, California, USA). Total RNA was isolated using TRIzol reagent (Invitrogen, California, USA) and cDNA was synthesized by reverse transcription of 1 μg of total RNA using iScript cDNA Synthesis kit (BIORAD, California, USA). For HOXD13, total RNA was isolated from limb bud of 4 day old control and treated embryos. Quantitative RT-PCR was performed using SYBR Select Master Mix (Applied Biosystems) in QuantStudio12K (Life Technologies) real-time PCR machine with primers (Table 1) to detect selected messenger RNA (mRNA) targets. The relative mRNA expression levels were normalized against expression levels of GAPDH for each sample and analyzed using 2^−∆∆CT^ method^51^.

**Table 1:** Primers used for RT-PCR.

Gene	Gene Name	Accession number	Primer Sequence (5′-3′)
GAPDH	Glyceraldehyde 3-phosphate dehydrogenase	NM_204305.1	F:-ACTGTCAAGGCTGAGAACGG R:-ACCTGCATCTGCCCATTTGA
HOXD13	Homeobox D-13	NM_205434.1	F:-TCTGGCTAATGGCTGGAACG R:-ATCTCGGGCTGGTTTAGTGC
CTNNB1	ß-catenin	NM_205081.1	F:-GTCCTGTATGAGTGGGAGCA R:-GTTTCGGGGAACATAGCAGAA
PITX2	Paired-like homeodomain 2	NM_205010.1	F:-CGATGAGTTGCATGAAGGAC R:-AGGAGGAAGGTGAGGAGGAG
LEF1	Lymphoid enhancer-binding factor 1	XM_015276137.1	F:-TCACCTACAGCGATGAGCAC R:-TATCAGGAGCTGGAGGATGC
WNT11	Wingless-type MMTV integration site family, member 11	XM_015280851.1	F:-TTCATCTTTGGCCCTGAATC R:-AGCTCGATGGATGAGCAGTT
PRKCA	Protein kinase C	XM_004946229.2	F:-ACAACCAGGACCTTCTGTGG R:-TCTCGTAGAGCAGCACTCCA
CAMK2D	Calcium/calmodulin-dependent protein kinase II delta	XM_015276279.1	F:-GCCAATCCACACCATTATCC R:-CCATCCATGTACTGCGTGAG
ROCK1	Rho-associated, coiled-coil containing protein kinase 1	XM_015277931.1	F:-TGACTGGTGGTCAGTTGGAG R:TAGAGATCTCGTTGTCATCAGG
ROCK2	Rho-associated, coiled-coil containing protein kinase 2	XM_015276085.1	F:-GACTGGTGGTCCGTAGGAGT R:-GCAGTCTCTCGGATGTTGTC

### Somite development

The embryos (three per group) were dissected from their membranes 24 hours after treatment (HH 23) and inspected using the dissecting microscope to count somite numbers.

### Statistical analysis

Data analysis was carried out by unpaired Student’s t-test or one way analysis of variance (ANOVA) using Graph Pad Prism 6.0 (CA, USA). Differences between control and treatment groups were deemed to be significant when *P* < 0.05.

## Electronic supplementary material

Supplementary file
