# Supplementary material for: TiO2 nanoparticles induce omphalocele in chicken embryo by disrupting Wnt signaling pathway
Source: Sci Rep. 2018 Mar 19;8:4756. doi: 10.1038/s41598-018-23215-7 (PMC5859020; doi:10.1038/s41598-018-23215-7)
Supplement: Supplementary file 1 — Supplementary file [file 41598_2018_23215_MOESM1_ESM.doc]

**TiO_2_ nanoparticles induce omphalocele in chicken embryo by disrupting Wnt signaling pathway**

**Shweta Patel^a^, Sarmita Jana^a^, Rajlakshmi Chetty^c^, Sonal Thakore^b^, Man Singh^c^ and Ranjitsinh Devkar^a*^**

^a^Department of Zoology, ^b^Department of Chemistry, Faculty of Science, The M.S. University of Baroda, Vadodara, India.

^c^School of Chemical sciences, Central University of Gujarat, Gandhinagar, India.

|  | **Author Name** | **Address and affiliation** | **Author detail** | **Email ID** |
| --- | --- | --- | --- | --- |
| 1 | Shweta Patel | Department of Zoology, Faculty of Science, The M.S. University of Baroda, Vadodara, India. | First author | patel_shweta27@yahoo.com |
| 2 | Sarmita Jana | Department of Zoology, Faculty of Science, The M.S. University of Baroda, Vadodara, India. | Co-author | sarmitajana@gmail.com |
| 3 | Rajlakshmi Chetty | School of Chemical sciences, Central University of Gujarat, Gandhinagar, India. | Co-author | rrlchetty2013@gmail.com |
| 4 | Sonal Thakore | Department of Chemistry, Faculty of Science, The M.S. University of Baroda, Vadodara, India. | Co-author | drsonalit@gmail.com |
| 5 | Man Singh | School of Chemical sciences, Central University of Gujarat, Gandhinagar, India. | Co-author | mansingh@cug.ac.in |
| 6 | Ranjitsinh Devkar | Department of Zoology, Faculty of Science, The M.S. University of Baroda, Vadodara, India. | *Corresponding author | phyto_met@yahoo.com |


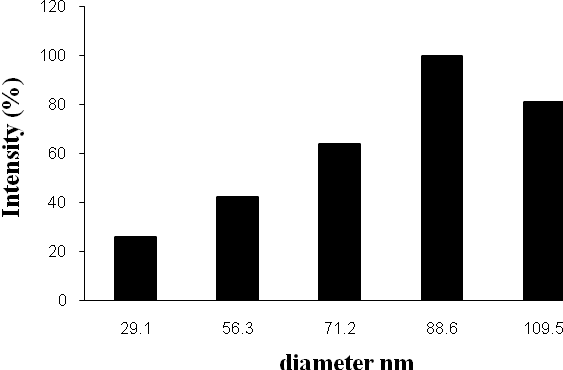


**Supplementary Figure S1**: Characterization of Titanium dioxide nanoparticles using dynamic light scattering

**Supplementary Table S2**: Microgram/microlitre (μg/ml), density (ρ ± 10^− 3^ kg m^−3^), viscosity (η ± 10^− 5^ mPa·s), surface tension (γ ± 10−2 mN m^−1^), friccohesity (σ, s.m.^−1^), activation energy (Δμ2^*^ ± 10^− 2^  kJmol^−1^), molecular radii (r, nm) at 37°C.

| µg/ml | ρ | η | γ | σ | Δµ_2_^*^ | r |
| --- | --- | --- | --- | --- | --- | --- |
| TiO_2_ NPs + Albumen | | | | | | |
| 1 | 1.031959 | 2.576379 | 65.45 | 0.002113 | -57.8093 | 5.425946 |
| 5 | 1.031876 | 2.604565 | 65.44 | 0.002136 | -57.9546 | 9.311949 |
| 10 | 1.031064 | 2.470162 | 65.98 | 0.00201 | -58.0253 | 11.52694 |
| 25 | 1.031567 | 2.492927 | 66.01 | 0.002027 | -58.5362 | 15.69238 |
| 50 | 1.031979 | 2.451386 | 66.64 | 0.001975 | -59.536 | 19.66072 |
| 100 | 1.032092 | 2.358271 | 66.65 | 0.001899 | -61.2601 | 24.45326 |

**Supplementary Table S3**: Percentage of secondary structure components in Native Albumen and TiO_2_ NPs + Albumen

| Wavenumber (cm^-1^) | % secondary structure | Secondary structural components |
| --- | --- | --- |
| **Native Albumen** | | |
| 1610 | 20.93 | Side chain |
| 1621 | 6.35 | Beta sheet |
| 1664 | 13.94 | Alpha helices |
| 1681 | 11.95 | Beta turn |
| 1695 | 10.76 | Beta sheet |
| 1629 | 32.76 | Beta sheet |
| **TiO_2_ + Albumen** | | |
| 1607 | 4.11 | Side chain |
| 1621 | 53.39 | Beta sheet |
| 1639 | 5.93 | Random coil |
| 1661 | 17.46 | Alpha helices |
| 1681 | 10.31 | Beta turn |
| 1695 | 8.81 | Beta sheet |

**Supplementary Table S4**: Organ weights of chicken embryos treated with Titanium oxide nanoparticles

| Organs | Control | Placebo | 10 μg/ml | 25 μg/ml | 50 μg/ml | 100 μg/ml | Bulk TiO_2_ | Cadmium chloride | p value |
| --- | --- | --- | --- | --- | --- | --- | --- | --- | --- |
| Number of embryos | 12 | 13 | 2 | 3 | 10 | 12 | 13 | 3 | - |
| Liver, g | 0.48±0.05 | 0.49±0.04 | 0.35±0.08 | 0.43±0.04 | 0.44±0.10 | 0.42±0.04 | 0.47±0.07 | 0.47±0.08 | ns |
| Brain, g | 0.43±0.10 | 0.40±0.04 | 0.42±0.07 | 0.34±0.03 | 0.39±0.07 | 0.31±0.12 | 0.41±0.09 | 0.39±0.07 | ns |
| Heart, g | 0.17±0.03 | 0.15±0.03 | 0.15±0.03 | 0.16±0.02 | 0.16±0.02 | 0.16±0.01 | 0.17±0.01 | 0.17±0.02 | ns |
| Spleen, g | 0.02±0.01 | 0.01±0.00 | 0.01±0.00 | 0.02±0.01 | 0.01±0.00 | 0.02±0.00 | 0.02±0.00 | 0.02±0.00 | ns |

Mean organ weights of embryos that were alive and without deformities. The control and groups treated with TiO_2_ NPs suspended in normal saline at concentrations of 10 -100 μg/ml. The data are expressed as Mean±SD. Statistical analysis was done by one way ANOVA. ns- not significant.
